# Evaluating the association of female obesity with the risk of live birth following IVF: Implications for clinical practice

**DOI:** 10.1093/humupd/dmz011

**Published:** 2024-01-16

**Authors:** Emma Schneider, Oliver Hamer, Chris Smith, James Hill

**Affiliations:** Liverpool University Hospitals NHS Foundation Trust; University of Central Lancashire; University of Central Lancashire; University of Central Lancashire

## Abstract

Obesity is a well-established risk factor for infertility. Consequentially, women living with obesity may require fertility treatment to support them to conceive. Due to evidence suggesting obesity is also linked with poorer outcomes following in vitro fertilisation (IVF), local commissioning guidelines on assisted conception recommend a BMI of <30kg/m^2^ before IVF can commence. However, it is currently unclear if these guidelines are evidence based. This commentary aims to critically appraise a recent systematic review by Sermondade et al, 2019 and expand upon the implications of the findings for clinical practice.

## Introduction

Obesity is increasing worldwide and the consequences in terms of its associations with morbidity and mortality have also been increasing ^1^. Obesity, defined as a Body Mass Index (BMI) ≥ 30kg/m^2^, is more common in women than in men ^1,2^. Estimates suggest that 19% of women of reproductive age in England are classified as obese ^2^. A BMI greater than 30kg/m^2^ is a well-established risk factor for infertility ^3^ and is associated with various reproductive sequelae including anovulation, subfertility, miscarriage, and poor neonatal and maternal pregnancy outcomes. ^1^ In addition, Polycystic Ovary Syndrome (PCOS), one of the most common endocrine conditions in female of reproductive age ^4^, is linked with both anovulatory infertility and obesity ^5,6^. As a consequence, women living with obesity may require fertility treatment to support them to conceive ^7^. One such strategy is in vitro fertilisation (IVF).

During IVF, female eggs (oocytes) are fertilized in a petri dish rather than in the ovary, which assists women who cannot conceive naturally ^8^. IVF is widely used internationally for the treatment of infertility from a range of causes, including endometriosis and unexplained infertility ^8^. Although there are no known contraindications of IVF, it has been suggested that the procedure should not be performed in patients who would have an increased risk of morbidity and mortality if IVF were successful (leading to pregnancy) ^8^.

There are several predictors of poorer pregnancy related outcomes following IVF, which include increasing female age, longer duration of subfertility, lower number of oocytes, decreased ovarian function and higher BMI ^9-11^. Recent evidence suggests that the factor of heighted BMI (i.e., obesity as defined by the WHO) is linked with poorer outcomes following in vitro fertilisation ^12-14^. This is reflected in policy as several NHS integrated care boards in England mandate (in their assisted conception policies) that patients have a BMI of below 30kg/m^2^ before IVF can commence ^15-17^. However, it is currently unclear if these guidelines are based on high quality and robust evidence. It is now important to synthesise existing evidence to establish if obesity is significantly associated with live birth rate following IVF. This commentary aims to critically appraise the methods used within the systematic review and meta-analysis by Sermondade et al, (2019), and explore its implications for clinical practice.

## Methods used by Sermondade et al. (2019)

The systematic review carried out a comprehensive multi-database literature search from 2007 to 2017, including databases such as PubMed, Embase, Cochrane Central Register of Controlled Trials, ClinicalTrials.gov, EU Clinical-trial register and Cochrane Database of Systematic Reviews ^18^. The systematic review protocol was registered on Prospero (CRD42018090645) and the review was reported in accordance with the PRISMA guidelines ^18^. There was a clear inclusion criteria which included cohort studies comparing IVF patients identified as obese (BMI ≥ 30 kg/m^2^ according to the World Health Organisation) versus “normal” weight (BMI 18.5–24.9 kg/m^2^) ^14,18^. The primary outcome of interest was live birth and studies were only included if they reported values of live birth for obese and “normal” weight females ^18^. There was also a transparent exclusion criteria stating that studies describing only women classified as overweight, underweight, or obese with another cut-off point other than BMI ≥ 30 kg/m^2^, were excluded. Studies were also excluded if they were reported as a conference abstract or clinical study, and the full text could not be retrieved.

Study selection and quality assessment (using the Newcastle-Ottawa Quality Assessment Scale) was undertaken independently by two reviewers. Any disagreements were discussed with a third reviewer until agreement was reached. Where appropriate, a random-effects meta-analysis (Mantel–Haenszel method) was undertaken using risk ratios with 95% confidence intervals (Review Manager 5.3.5). A funnel plot was employed to assess publication bias. Heterogeneity across the studies was judged by the value of the I^2^ statistics. Subgroup analyses was performed to distinguish between distinct kinds of embryo transfer, cycle rank of the IVF, oocyte source and patients diagnosed with PCOS. Sensitivity analysis was conducted by excluding all studies with at least one high risk of bias, and any outliers identified in the funnel plot.

## Results

A total of 48 studies were included in the review of which 21 case studies were meta synthesised. The majority of the 21 case studies were undertaken within the United States (n= 13) with the remaining studies being carried out in France, Denmark, Spain, Macedonia, Australia, China, and India. Of these 21 studies, the three main areas of risk of bias (high risk of bias/clear) were bias due to confounding (n =14), bias in classification of interventions (n = 7) and bias in selection of participants into the study (n = 6). A sensitivity analysis of only those studies which had at least one criterion at high risk of bias (this did not include studies where the bias was classified to be unclear), showed no statistically significant difference in relative risk of live birth rate (visual inspection) ^18^.

When meta synthesised there was a statistically significant reduction in risk of live birth comparing women with BMI ≥ 30 kg/m^2^ to women with a BMI in 18.5–24.9 kg/m^2^ (Risk Ratio [RR] 0.85, 95% CI: 0.82–0.87; moderate heterogeneity). There was also a statistically significant reduction in risk of live birth for women with a BMI a 25.0–29.9 kg/m^2^ compared to a BMI 18.5–24.9 kg/m^2^ (RR 0.94, 95% CI: 0.71–0.97; moderate heterogeneity).

A range of subgroup analyses were undertaken to identify possible important moderating factors. On visual inspection there was no evidence that the relative risk of live birth changes based upon cycle rank when comparing women with BMI ≥ 30 kg/m^2^ to women with a BMI in 18.5–24.9 kg/m^2^ (only first cycle, all cycles, unspecified). The subgroup analysis exploring ovarian status found a statistically significant reduction in relative risk of live birth for women with PCOS with a BMI ≥ 30 kg/m^2^ compared to a BMI 18.5–24.9 kg/m^2^ (RR 0.78, 95% CI: 0.74–0.82, no unexplained heterogeneity). There was no evidence of difference between women with without PCOS with a of BMI ≥ 30 kg/m^2^ compared to a BMI 18.5–24.9 kg/m^2^. Due to a lack of studies the subgroup analysis for embryo transfer type was unable to be compared.

## Commentary

The AMSTAR-2 critical appraisal tool for systematic reviews was employed to assess the methodological quality of the review by Sermondade et al, 2019 ^19^. The AMSTAR-2 tool was chosen because it is widely considered to be a comprehensive, valid, and reliable tool for assessing the quality of systematic reviews ^20^.

Of the 16 AMSTAR-2 criteria, 14 were met, indicative of a robust and comprehensive summary of evidence. Two criteria were not met as the study did not provide a list of excluded studies or justify the exclusions and did not disclose any competing interests of the authors. A further concern was the high heterogeneity observed in the analysis which may increase the risk of bias. Variability within the study population increases the difficulty to detect true associations or effects because it reduces statistical power ^21^. In addition to these concerns, the date of search (2017) could be considered outdated, and this may result in more recent relevant studies not included within the analysis. A further concern was that this systematic review did not undertake a meta-regression to explore the possible cause of the moderate heterogeneity observed in the main comparison. This makes it difficult to identify what possible moderating factors may influence the effect such as study location and age of participants.

One of the main limitations of this systematic review is how applicable the findings are to clinical practice. Notably, there is no comparison of live birth rates between women who are classified as obese (BMI ≥ 30 kg/m^2^) and women who are classified as overweight (25 to 29.9 kg/m^2^). This is because the main analysis only compared women who were classified as either overweight or obese against women classified as ‘normal weight’ (BMI 19 to 24.9 kg/m^2^). As IVF in England is often limited to women who are classified as overweight or a healthy weight ^15-17,22^, it would be useful to determine whether women with a BMI ≥ 30 kg/m^2^ had a significant decreased chance of giving birth following IVF when compared with women with a BMI <30 kg/m^2^. A further limitation is that the article does not use people-first language and describes the population group as ‘obese infertile women’. In addition, the article also describes the women with a BMI of 18.5kg/m2-24.9kg/m^2^ as a ‘normal weight’ rather than as a healthy weight as per NICE guidance. The absence of people-first language may lead to the bias and discrimination of people living with obesity; undermining the quality of the study. Another key limitation is the lack of clarity as to whether patients with a BMI of <30 kg/m^2^ have undergone weight reduction. From a clinical perspective, it is the important to establish the effects of those who have not undergone a weight reduction program compared to those who have. As a consequence, the population in the study may be deemed to have reduced indirectness regarding this clinical scenario.

Within the subgroup analysis, the review only included the 4 studies which were classified to have a high risk of bias for one criterion ^18^. Subsequently, the review did not assess the possible effect of the 14 studies with unclear classification of at least one category of bias. As a consequence, it is unclear what effect these issues of bias may have had on the relative risk of live birth following IVF. Despite the above limitations, the review provides a comprehensive and complete summary of the evidence of interest. However, it is important consider these methodological issues when interpretating the findings, as they may reduce the certainty of the effect estimates, and external validity of the findings.

The findings from the review indicate that there may be a clinical and statistically significant decreased risk of live birth following IVF comparing women with a BMI of ≥ 30 kg/m^2^ to 18.5–24.9 kg/m^2^. Furthermore, there was a significant decreased risk of live birth comparing women with a BMI of 25.0–29.9 kg/m^2^ to 18.5–24.9 kg/m^2^. It is important to note when interpreting the findings that there was moderate unexplained heterogeneity which would reduce the certainty within the estimates. These findings do suggest that BMI of ≥ 30 kg/m^2^ may negatively impact live birth rates following IVF. However, it is still unclear at what BMI threshold the risk may substantially reduce as no direct comparison was made between BMI of ≥ 30 kg/m^2^ and BMI of 25.0–29.9 kg/m^2^. As mentioned above, a clinically important comparison which was not explored, would be those who have a BMI of ≥ 30 kg/m^2^ and those who previously had a BMI of ≥ 30 kg/m^2^ and have now lost the weight. The findings also showed that there was some evidence that PCOS may be an important moderating factor.

## Conclusion

There are numerous confounding factors which are potentially associated with obesity and fertility, including exercise, dietary patterns, alcohol intake, stress and smoking ^3,23,24^. These confounding factors were not considered in the meta-analysis and systematic review by Sermondade et al, 2019. In clinical practice, utilising a range of lifestyle screening tools such as the recently developed nutrition screening tool for dietetic intervention ^25^, may provide a more holistic approach to identifying and optimising these lifestyle factors, rather than using BMI as a binomial cut-off. Based upon this possible multifactorial effect in risk, it may be proposed that a weighted model for each individual risk factor may be more appropriate.

As highlighted above there is a need for a further meta-analysis comparing the effects of women with a BMI ≥ 30 kg/m^2^ compared to BMI <30 kg/m^2^ (specifically in the overweight BMI range) on probability of live birth following IVF. Furthermore, further research should examine the probability of live birth following IVF for those who have gone through a weight reduction program in groups with a BMI <30 kg/m^2^ compared to women with a BMI ≥ 30 kg/m^2^. Additionally, research should explore the exact mediating factors of any potential change in risk associated with BMI and outcomes relating the IVF. Finally, as the review by Sermondade et al is somewhat out of date, it is recommended that an update of this review is undertaken.

Practise challenge questions

1. What are the limitations and strengths of the evidence synthesised by the systematic review?
2. What are the limitations of a BMI of 30kg/m^2^ as an eligibility threshold for IVF treatment?
3. What are limitations of solely relying on BMI to define obesity?

## Figures and Tables

**Table 1:** Critical appraisal using the AMSTAR-2 tool for assessing systematic reviews

AMSTAR 2	Responses
1. Did the research questions and inclusion criteria for the review include the components of PICO?	Yes – The study included all components of PICO. Females, BMI ≥ 30 kg/m^2^ Cohort studies comparing IVF patients Healthy weight females Live birth was the outcome
2. Did the report of the review contain an explicit statement that the review methods were established prior to the conduct of the review and did the report justify any significant deviations from the protocol?	Yes – The search strategy, selection criteria, data extraction, quality assessment and statistical analyses described below were defined a priori
3. Did the review authors explain their selection of the study designs for inclusion in the review?	Yes - The study outlines the use of cohort studies
4. Did the review authors use a comprehensive literature search strategy?	Yes – A comprehensive search strategy with appropriate MeSH terms and keywords was included.
5. Did the review authors perform the study selection in duplicate?	Yes - Two reviewers independently performed study selection. However, the two reviewers’ professional involvement was not explained. Additionally, there was no indication of what the process that included a third reviewer included.
6. Did the review authors perform data extraction in duplicate?	Yes - Two reviewers conducted data extraction from included studies
7. Did the review authors provide a list of excluded studies and justify the exclusions?	No –information was not included in the publication or supplementary information
8. Did the review authors describe the included studies in adequate details?	Yes - Each included paper was detailed in the characteristics of included studies table (table 1).
9. Did the review authors use a satisfactory technique for assessing the risk of bias in the individual studies that were included in the review?	Yes - Review authors used a risk of bias tool which included appropriate domains. The RoB assessment is seen in figure 2.
10. Did the review authors report on the sources of funding for the studies included in the review?	Yes -The RoB assessment included funding from each study and the review was sponsored by an unrestricted grant from GEDEON-RICHTER France.
11. If meta-analysis was performed did the review authors use appropriate methods for statistical combination of results?	Yes - Authors used random effects model, risk ratios, Chi^2^ and I^2^ values for meta-analysis heterogeneity.
12. If meta-analysis was performed did the review authors assess the potential impact of RoB in individual studies on the results of the meta- analysis or other evidence synthesis?	Yes - Pooled estimates were based on the studies and an analysis was performed on possible impact of the bias.
13. Did the review authors account for RoB in individual studies when interpreting/discussing the results of the review?	Yes - When there was moderate to high risk of bias the review included discussion on impact and also excluded study of high risk of bias in separate analysis
14. Did the review authors provide a satisfactory explanation for and discussion of, any heterogeneity observed in the results of the review?	Yes - Where heterogeneity existed, the authors provided an investigation for sources of heterogeneity and concluded that it prevents drawing firm conclusions from the data.
15. If they performed quantitative synthesis did the review authors carry out an adequate investigation of publication bias (small study bias) and discuss its likely impact on the results of the review?	Yes - A funnel plot was used to assess the presence of small-study effects suggestive of publication bias
16. Did the review authors report any potential sources of conflict of interest, including any funding they received for conducting the review?	No – Competing interests were not outlined in the publication or supplementary information, but they did state the funding received (grant from GEDEON-RICHTER France).

## Key Findings

- A decreased probability of live birth following IVF was observed in women with obesity when compared with women who are a healthy weight.
- There may be a decrease probability of live birth following IVF in women who are overweight compared to women who are a healthy weight.
- There was no evidence that the relative risk of live birth changes based upon IVF cycle rank when comparing women with obesity to women who are a healthy weight.
